# A New Method for the Production of High-Concentration Collagen Bioinks with Semiautonomic Preparation

**DOI:** 10.3390/gels10010066

**Published:** 2024-01-15

**Authors:** Jana Matejkova, Denisa Kanokova, Monika Supova, Roman Matejka

**Affiliations:** 1 Department of Biomedical Technology, Faculty of Biomedical Engineering, Czech Technical University in Prague, 272 01 Kladno, Czech Republic; kanokden@fbmi.cvut.cz; 2 Department of Composites and Carbon Materials, Institute of Rock Structure and Mechanics of The Czech Academy of Sciences, v.v.i., 182 09 Prague, Czech Republic; supova@irsm.cas.cz

**Keywords:** bioprinting, bioink, collagen hydrogels, biofabrication, pH, neutralization, automation, stromal cells

## Abstract

It is believed that 3D bioprinting will greatly help the field of tissue engineering and regenerative medicine, as live patient cells are incorporated into the material, which directly creates a 3D structure. Thus, this method has potential in many types of human body tissues. Collagen provides an advantage, as it is the most common extracellular matrix present in all kinds of tissues and is, therefore, very natural for cells and the organism. Hydrogels with highly concentrated collagen make it possible to create 3D structures without additional additives to crosslink the polymer, which could negatively affect cell proliferation and viability. This study established a new method for preparing highly concentrated collagen bioinks, which does not negatively affect cell proliferation and viability. The method is based on two successive neutralizations of the prepared hydrogel using the bicarbonate buffering mechanisms of the 2× enhanced culture medium and pH adjustment by adding NaOH. Collagen hydrogel was used in concentrations of 20 and 30 mg/mL dissolved in acetic acid with a concentration of 0.05 and 0.1 wt.%. The bioink preparation process is automated, including colorimetric pH detection and adjustment. The new method was validated using bioprinting and subsequent cultivation of collagen hydrogels with incorporated stromal cells. After 96 h of cultivation, cell proliferation and viability were not statistically significantly reduced.

## 1. Introduction

Bioinks—polymers with incorporated cells ‘customized’ for the preparation of tissues and organs—must meet many criteria to be structurally strong enough for the preparation of 3D structures, but also non-toxic to organisms, especially the cells that populate the gel. Ideally, cell adhesion and proliferation are also promoted. The resulting bioink should be nonimmunogenic and biodegradable [1]. Using natural or synthetic polymers is an option, where, in addition to properties, we must also consider the processability of the material, suitability for bioprinting, cost, and commercial availability. While synthetic polymers excel in mechanical durability, natural gels are highly biocompatible [2]. Using a natural material with a high concentration of polymers combines the advantages of both types of materials but has its limits for adhesion and cell growth [3].

A suitable natural polymer for bioink preparation is collagen, which is abundant in the extracellular matrix and thus is very natural to use and is also highly available in the connective tissues of organisms [4]. Collagen has a high affinity for adherent cells, which is the reason why these hydrogels are widely used in biomedical applications, from testing materials for 3D bioprinting to general tissue models for in vitro cell studies, drug testing, and specialized tissue models, especially when using cell-filled hydrogels [5].

Due to its good biocompatibility and low immunogenicity, collagen has been successfully used in clinical practice. However, low immunogenicity can only be achieved using high-purity collagen solutions when the protein is derived from collagen-containing tissues. The main weaknesses of this material are its low mechanical properties, difficulty in sterilization (e.g., heat sensitivity, degradation), and the commonly occurring shrinkage of collagen scaffolds in response to cellular activity [5]. Regarding 3D printing applications, collagen-based bioinks with potential for tissue regeneration have been developed to produce breast implants and artificial cartilage for joint reconstruction, among others [6]. Cell-based collagen bioinks also contribute to the growth of the 3D-printed alternative meat market, which companies are exploiting [7].

However, in the present studies, relatively low-concentration collagen polymers are used, as higher concentrations (10 mg/mL) reduce cell proliferation in the gel and differentiate ability, and the diffusion of waste products in the polymer is also impeded [8,9]. However, several factors influence the successful formation of the 3D structure. These include the properties of the bioink itself (composition, pH, temperature, viscosity, cell culture concentration, and crosslinking method) as well as the parameters of the printing process (temperature, pressure, and speed of parts of the printing system parts), as they have a significant impact on the resulting structural strength and viability of the incorporated cells [5].

Collagen hydrogels (COLs) are usually created from cold, acid-solubilized monomers (tropocollagen) by neutralization and heating to induce physical crosslinking. COL gelation is due to fibrillogenesis, a self-assembly process that proceeds from the self-association of triple helices [10]. This process is strongly affected by the nature of the COL monomers and many other physical factors [11]. Fibrillogenesis is induced by the neutralization of pH and by increasing temperature while increasing the ionic strength, which causes a decrease in the rate of fibrillogenesis [12]. The kinetics of COL fibrillogenesis consist of two stages: (1) a nucleation process and (2) the subsequent growth of nuclei and the aggregation of COL fibrils that result in the 3D network structure of the hydrogel [13]. Both the pH and the ionic strength affect the net surface charges of the COL molecules. Low pH causes a repulsive electrostatic interaction between COL molecules. Consequently, they are homogeneously dissolved [14], while neutral pH conditions cause a weakening of electrostatic repulsion. The kinetics of fibril self-assembly are also affected by the alteration of electrostatic, hydrophobic, and covalent interactions between monomers, resulting in the range of fibril size [11] and the final microstructure and the properties of the collagen gel [15,16]. These interactions are strongly influenced by concentrations of both COL and solvent [17]. The highest rates of fibrillogenesis occur between pH 6.9 and 9.2, with no significant changes in fibril diameters [18]. Another important factor is temperature, which affects the water-mediated hydrogen bonding between collagen molecules because water between triple helices may play a role in the association process [19].

Gelation occurs in two stages: first, triple helices in COL molecules are dissolved in dilute acid (typically acetic acid for several days at low temperatures) to disrupt weaker intermolecular hydrogen bonds and Schiff bases between collagen molecules [20], followed by the formation of 3D hydrogel structures induced by increasing the temperature and pH value (using dilute alkali solution or various simulated body fluid media). Therefore, COL hydrogel formation is a process of reconstruction of collagen molecules with water in a 3D space.

Studies that have used collagen concentrations of 10 mg/mL or more for bioink preparation have often used additives (riboflavin, Pluronic F127) to crosslink the polymer, resulting in increased mechanical strength of gels [21]. Still, the substances used are unnatural to cells. They can interfere with cell proliferation and differentiation, as well as the biocompatibility of the entire structure [22]. Crosslinking by mere modification of physical conditions (temperature, pH) is rarely represented due to high collagen concentrations [9].

In our previous work [23], we optimized the bioink properties with highly concentrated collagen (20 mg/mL dissolved in 0.1% acetic acid; the final collagen concentration was 10 mg/mL), especially the gel composition and adjustment of pH. When NaOH was added, we adjusted the final pH and gelation time. The composition and pH adjustment allowed the cells to grow and divide, and the higher mechanical modulus of these constructs also allowed the structures to be printed up to several millimeters in height with sufficient mechanical resistance. This experiment demonstrated that a high concentration of collagen gels is not necessarily a limiting factor for cell proliferation.

However, from an objective point of view, higher cell viability could be achieved in the resulting structures. Furthermore, the bioink preparation method also needed to be optimized regarding component volumes, as they varied up to a thousand times (from a few µL to several mL). This is particularly disadvantageous for mechanized, automated bioink preparation. Therefore, our paper aims to eliminate these weaknesses and obtain a 3D culture with high cell viability even after several days of cultivation.

This study builds on the results of a previous study to optimize the bioink composition so that cells achieve higher viability and proliferation even after several days of static culture. Furthermore, we also want to improve the method of mixing the bioink so that higher volumes of components can be handled, and a machine can then implement the mixing.

## 2. Results and Discussion

### 2.1. Cell Growth in Modified Culture Media

New formulations of culture media with a compensated additive concentration were compared to the formulation from our previous article and control growth culture medium. Growth and viability were evaluated after 3 days of static cultivation.

**Figure 1:**
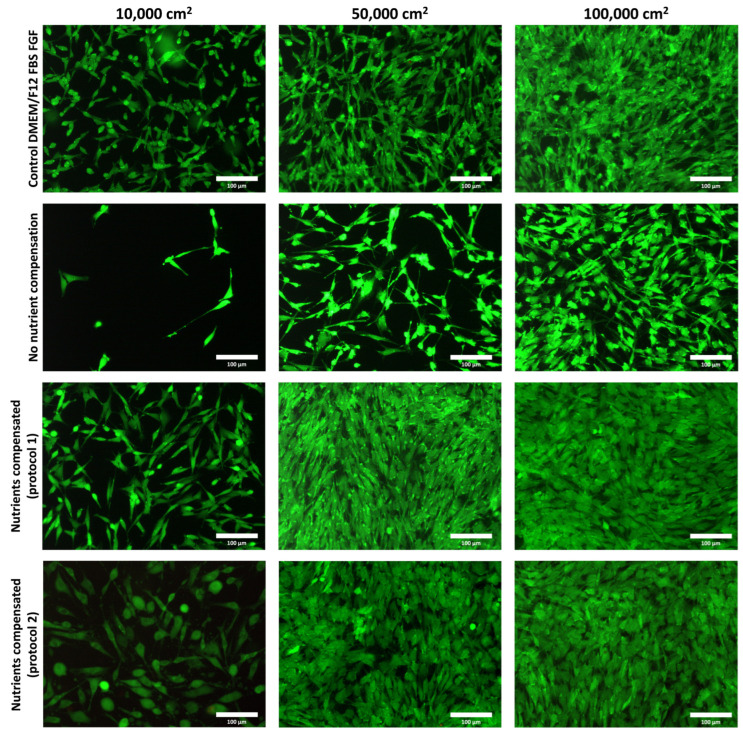
Live/dead images of cells cultured for 3 days in control culture medium, without compensation of nutrients and compensated (protocol 1, e.g., pH adjusted culture medium, and protocol 2, culture medium without pH adjustment). Live cells were stained with fluorescein diacetate (green), and dead cells (nuclei) were stained with propidium iodide (red), scale bar 100 µm—custom-built Thorlabs CERNA fluorescence microscope with Olympus Plan Fluorite 10× objective.

Cells in the nutritionally compensated media have proliferation similar to that of the control sample, although their shape is slightly elongated. In an uncompensated medium, proliferation is lower in all hatches. In this medium, the cells are not elongated are more isolated, and do not form a continuous structure as in the other samples. This indicates insufficient cell proliferation, which does not result in adequate formation of the potential tissue base. As illustrated in Figure 1, a medium lacking nutrients degraded cell growth.

Furthermore, nutrition-compensated media were tested to determine whether high concentrations of medium components during bioink preparation would result in cell damage and thus reduce viability. After 3 days of culture, there was no significant difference from control cells grown in the original culture medium. No dead cells are visible in any of the images; as in these 2D cultures, the cells are rinsed with PBS before analysis, and thus, the dead cells are removed from the culture.

### 2.2. Collagen FTIR Spectra

**Figure 2:**
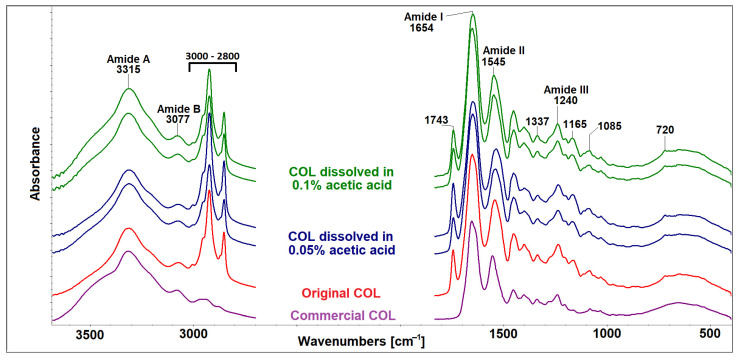
FTIR spectrum of porcine collagen lyophilizate after isolation (original COL) and examples of spectra of COL after dissolution in 0.05 wt.% and in 0.1 wt.% acetic acid in a COL concentration of 20 mg/mL and comparison with commercial COL.

The FTIR spectra of isolated COL (original) and COL dissolved in 0.05 wt.% and in 0.1 wt.% acetic acid in a COL concentration of 20 mg/mL can be seen in Figure 2. All spectra contained five amidic bands typical for proteins. The band cantered at 3315 cm^−1,^ relating to a mutual band of hydrogen bonds among OH groups of water and humidity and amide A of COL, which belong to NH_2_ stretching. The band at ~3077 cm^−1^ is mutual band of the N-H stretching vibrations in the secondary amides (amide B) and C–H stretching vibrations in the sp2 hybridization existing in aromatic amino acids. The amide I band describes the C=O stretching coupled with N–H bending vibrations. The amide II band originates from N–H bending coupled with C–N stretching vibrations. Another proof of the existence of a triple helical structure in the COL represents a band triplet (at ~1205, 1240, and 1280 cm^−1^) of amide III together with the band at 1337 cm^−1^ [24,25]. Changes in the intensities and positions of individual amide bands could be connected with changes in the secondary structure of COL. As was proved in our previous study [23], the use of 70% ethanol for sterilization did not alter the secondary structure of COL. As shown in Figure 2, the use of acetic acid as a solvent, regardless of concentration, does not change the secondary structure of COL compared to original and commercial COL.

Apparent changes in band intensities in spectral region 2800–3000 cm^−1^ and at 720 cm^−1^, relating to C-H aliphatic bonds, and changes in bands at 1743 cm^−1^, 1165 cm^−1^ and 1085 cm^−1^ ascribed to the C=O bonds in ester, are related to the presence of lipids. The different intensities of the infrared bands belonging to lipids in all spectra are caused by local inhomogeneity. Lipids are common residual impurities remaining in collagen after the isolation procedure [24]. As can be seen in Figure 2, the commercial COL does not contain such impurities.

The comparison of the infrared spectra of the original collagenous lyophilizate and the collagens subjected to acetic acid of two various concentrations (0.05 wt.%, 0.1 wt.%) proved that the spectra following dissolution in acetic acids did not exhibit significant changes compared to original COL.

### 2.3. Effect of Medium Preconditioning

**Figure 3:**
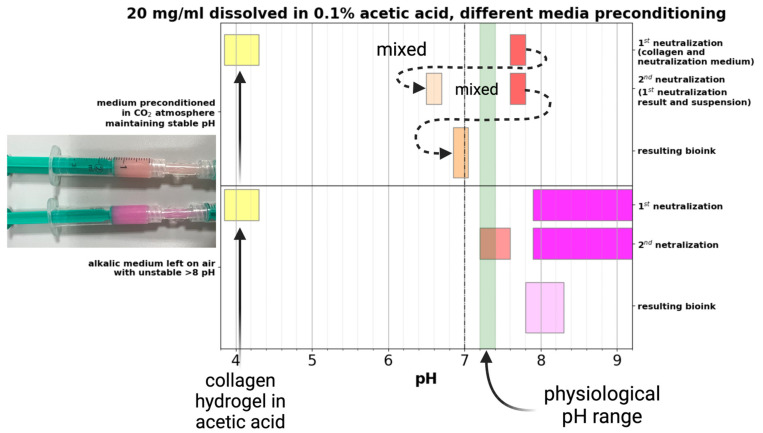
The difference in the pH of the resulting bioink is affected by the initial condition of the culture medium preconditioned in a CO_2_ atmosphere and left in the air. Created with BioRender.com.

The culture media used in this study use a bicarbonate and CO_2_ buffering system that affects the resulting pH [26]. In our case, we prepared 2× enhanced media that is in an optimal state above the physiological range of approximately 7.6–7.8, depending on the batch and adjustments before sterilization. Figure 3 illustrates different pH levels of culture media preconditioned in a CO_2_ atmosphere for 120 min and media left on air. The medium left in the air, due to the lack of CO_2_, changed to an alkaline pH. The range of change was affected by the length of exposure, its volume, and also by the fact that our “2× enhanced” medium contains twice the bicarbonate compared to the standard proliferation medium (thus the large interval in the chart). The primary challenge is that the pH level of the culture medium has an impact on the neutralization of the collagen hydrogel. The pH of the collagen hydrogel then affects the gelling properties, cell viability, and printability. To ensure consistency and reproducible outcomes, we have used only preconditioned media in the following preparations of printable bioinks.

### 2.4. pH Response of Collagen Hydrogel to Medium Added

**Figure 4:**
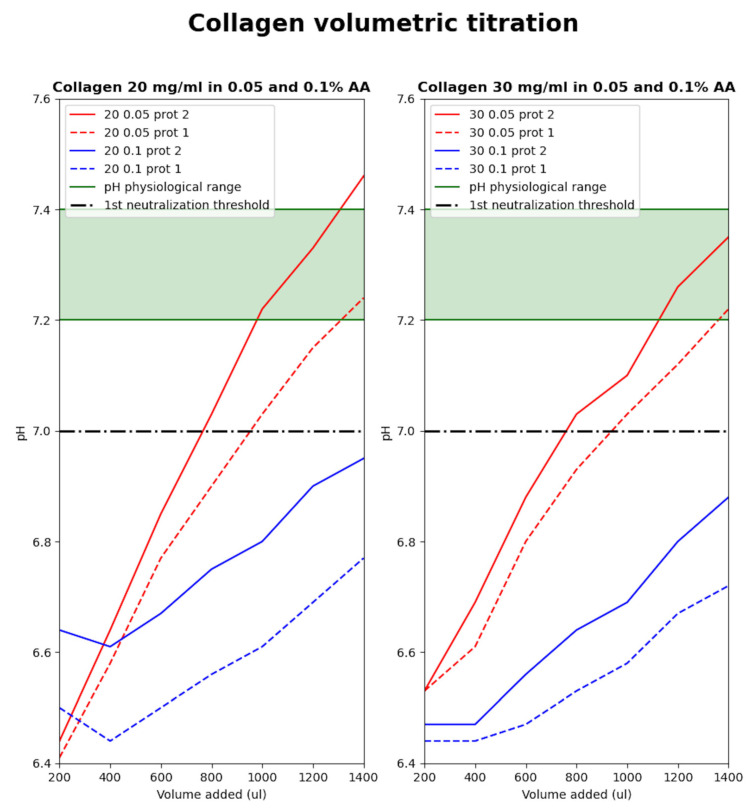
Volumetric titration of collagen hydrogels and response to changes in pH.

As described above, the use of lower concentrations of acetic acid (AA) in the dissolution of collagen yields almost an optimal pH after neutralization. Furthermore, some different adjustments of the culture media prior to sterilization (especially 10× concentrated) can affect the resulting parameters. To demonstrate this, we performed a simple volumetric neutralization in our mixing system. The mixing syringe was prefilled with 1400 µL of collagen (all variants—concentration of collagen 20 and 30 mg/mL and 0.05 and 0.1% dissolving AA). Then, the ‘2× enhanced’ medium was dosed in 200 µL steps. After each addition, the collagen and the media were mixed, and the pH was captured using a colorimeter. This was performed until 1400 µL of media was dosed to achieve a 1:1 ratio of collagen to medium. As illustrated in Figure 4, it is obvious that 0.1% AA provides a more acidic and probably buffered collagen hydrogel, which may require more culture medium or NaOH modification. Differences in the preparation of the culture medium and its adjustments before sterilization can affect the neutralization behavior. In the case of 0.05% AA, there is a promising fact that with a different approach in media preparation, there is no need to perform any NaOH modification in culture media. Another approach can utilize continuous mixing and the estimation of pH in a designed mixing system, allowing for the change in the ratio between the neutralization and cell suspension media.

In our described experiments, the neutralization medium was modified using fixed steps of NaOH and a fixed 2:1:1 ratio (collagen:neutralization medium:suspension medium). These optimizations will be made in the following research.

Sterilization of the medium by filtration causes an increase in pH, and titration with this medium leads to an optimal pH much earlier. Collagen dissolved in less concentrated acid reaches physiological pH values with the addition of approximately 1 mL of culture medium so that further pH adjustment with NaOH is no longer necessary. This confirms the results of the previous analysis. By selecting the appropriate concentration of solvent and neutralizing with 2× enhanced medium, the pH of the mixture reaches a value of 7, at which point the cell suspension can be added without negatively affecting cell proliferation or viability. In some cases, it is unnecessary to add NaOH to the mixture, as we have implemented and published the results of the previous article [23] so far.

### 2.5. The pH Balancing

**Figure 5:**
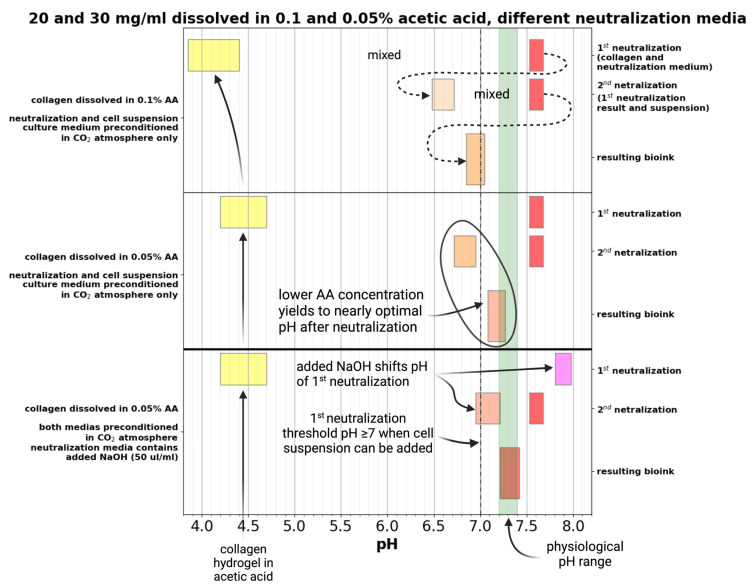
Two-step neutralization of collagens dissolved in 0.1 and 0.05 wt.% acetic acid using preconditioned and NaOH-modified 2× enhanced culture medium. A lower concentration of acetic acid provides a nearly optimal pH of the resulting bioink. However, depending on the batch of collagen, other pH adjustments using NaOH are optimal. Created with BioRender.com.

The pH of the prepared bioink varies according to the concentration of AA in which collagen dissolves, as shown in Figure 5. In the case of 0.1% acid, after the first neutralization, the pH is around 6.5, which is a fairly acidic environment for adding cells. After the second neutralization, the pH is below the physiological limit. Using a less concentrated solvent (0.05 wt.% AA) and no NaOH, the pH values are higher than with 0.1% acid but still below the physiological limit. The addition of NaOH at a concentration of 50 µL/mL leads to a change in pH below the physiological limit after the first neutralization. After the second neutralization, the pH is fully physiological for cell development.

**Figure 6:**
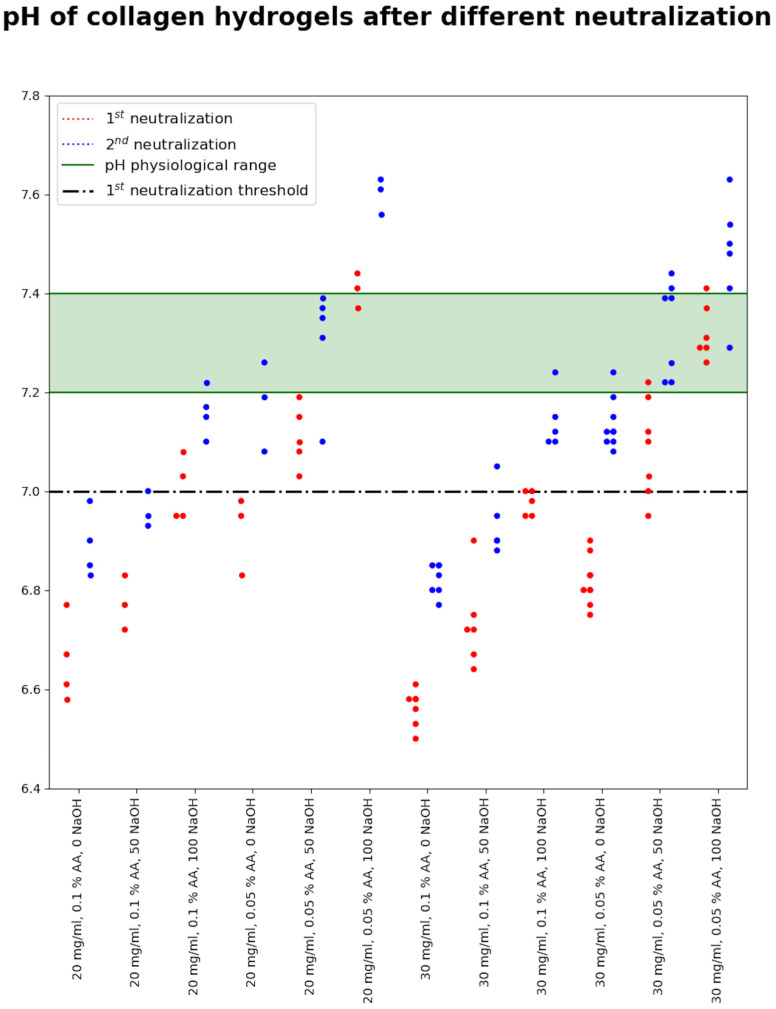
pH of the bioinks prepared after the first and second neutralization using a different neutralization medium (different concentrations of NaOH added).

To find the optimal neutralization medium and procedure, we find that two concentrations of collagen (20 and 30 mg/mL) and both dissolving AA concentrations (0.05 and 0.1 wt.%) were first neutralized using 2× enhanced medium with 0, 50, and 100 µL/mL NaOH. This initial neutralization step is crucial because it shifts the pH level closer to the physiological range, thereby making it more compatible and acceptable for cells. This prevents the cells from experiencing a shock from a rapid change in pH, which results in higher cell viability. The second neutralization step was performed with a 2× enhanced culture medium. The resulting pHs are illustrated in Figure 6. The 0.1% AA in both concentrations of collagen is obviously more acidic than 0.05%. Even when 100 µL/mL NaOH was used, collagens dissolved in 0.1% acidic were below the physiological range of pH. The 0.05% acidic acid as the dissolving agent provided easier control over the pH range. When neutralized without adding NaOH, the resulting pH after the first neutralization was slightly above 7 (6.8–7) and after the second neutralization reached the physiological range. However, it was still slightly below 0.1–0.15. For the following prints with living cells, 50 µL/mL NaOH was chosen as the optimal amount to add.

**Figure 7:**
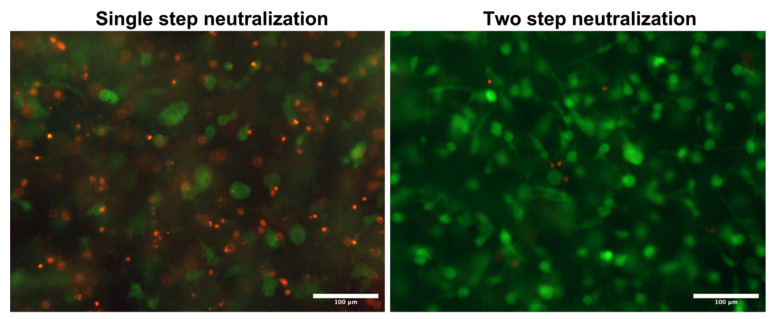
Difference in cell viability using single- or two-step neutralization. Live/dead images of cells grown for 3 h in a printed collagen bioink. Live cells stained with fluorescein diacetate (green) and dead cells (nuclei) stained with propidium iodide (red), scale 100 µm. Custom-built Thorlabs CERNA fluorescence microscope with Olympus Plan Fluorite 10× objective.

We have used two-step neutralization, where first neutralization shifts the pH of acid collagen into a natural pH of around 7. In addition, we chose this value as the working threshold for the second neutralization with culture media. As a simple alternative, we tested only single-step neutralization. In single-step neutralization, we mixed both neutralization media containing NaOH and cell suspension together. Then, it was mixed with acid collagen. The resulting pHs were similar to those of two-step neutralization; however, it resulted in poor cell viability. Skipping the two-step neutralization procedure leads to apoptosis of most cells in the hydrogel, as demonstrated in Figure 7.

Although it must be taken into account, the use of a less concentrated acid can result in less pH fluctuation during bioink preparation, and it can also not completely dissolve collagen fibers, which would have an adverse effect on subsequent printing.

In a previous article, we determined the optimal volumetric concentration of NaOH in collagen bioink to be 20 mg/mL in 0.1% AA–10 µL/mL, in terms of both mechanical strength and cell survival. Now, we must refine this conclusion. Several circumstances influence the resulting pH. Not only is the concentration of collagen in the bioink and the concentration of acid used for dissolution, but the pH is slightly different in each batch of production as a result of natural influences. Thus, it is not possible to generally determine the appropriate concentration of hydroxide to stabilize the pH, but in each individual experiment, the pH must be monitored and adjusted, if necessary, before cell suspension.

### 2.6. Cellular Behavior in Bioinks

**Figure 8:**
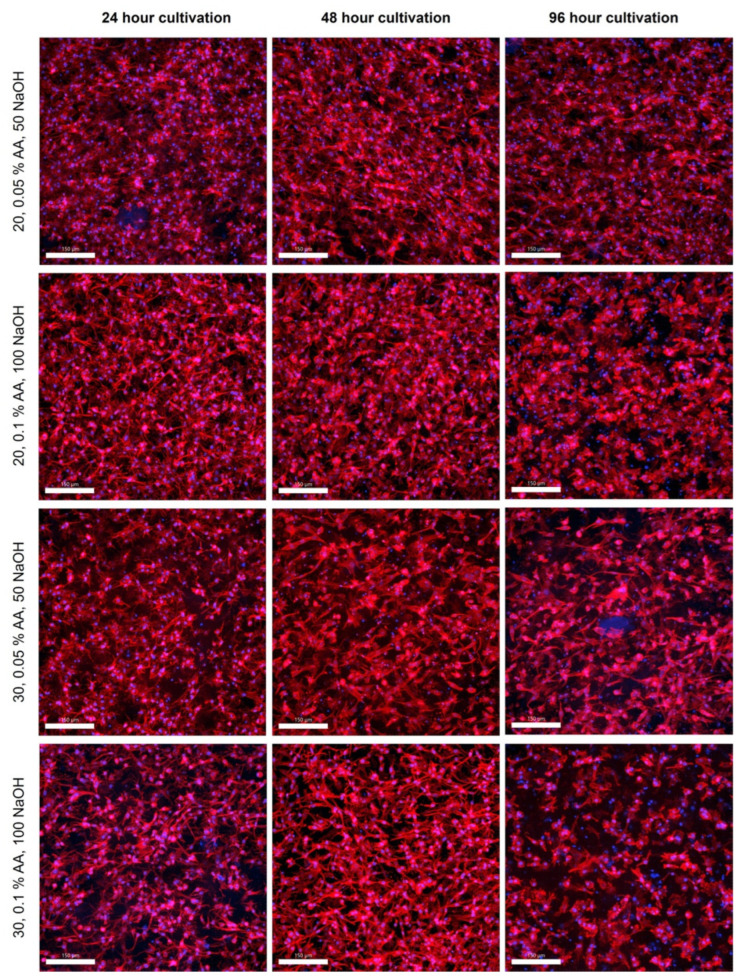
Confocal microscopy images of cultivated samples in 20 and 30 mg/mL collagen dissolved in 0.05 and 0.1 wt.% acetic acid. Cultivation for 24, 48, and 96 h. F-actin was stained using Alexa Fluor™ 594 Phalloidin (red), and cell nuclei were counterstained using DAPI (blue), scale 150 µm. Nikon CSU-W1 confocal microscope with CFI Plan Apo VC 20× dry objective. Orthographic projection rendered.

It is well known that high-concentrated collagen hydrogels, especially their stiffness, porosity, pH level, chemical composition, and presence of adhesive ligands, affect cell morphology and behavior [27]. We analyzed the cell morphology within the four different categories of gels at three different time points. The stromal cells in the hydrogel showed high viability and density even in the inner parts of the materials after 96 h of culture, as shown in the confocal microscope images in Figure 8. For samples with different concentrations of AA, no difference in cell viability was evident because the gels were prepared by two-step neutralization, where the cell suspension was mixed into a gel at a pH of approximately 7. A slight but not statistically significant difference was observed for samples with different concentrations of 20 and 30 mg/mL (resulting in concentrations of 10 and 15 mg/mL). The 30 mg samples, regardless of the concentration of AA, showed lower cell viability at some time intervals. With the more concentrated collagens, the hydrogel does not mix completely with the cells, and the cells form clusters where more cells are clumped, so the gel is not completely uniformly populated with cells. Thus, these clusters are more susceptible to automatic and manual cell counting. Inaccuracies in counting also introduce some errors, as the cells are interspersed throughout the gel volume, and cell viability varies greatly from each section.

This confirms the validity of our method of gradual neutralization of collagen hydrogels during bioink preparation. None of the samples show reduced cell proliferation. This corresponds to our results that, depending on the actual batch and acidity of the collagen used, the process method needs to be adjusted to optimize the pH to physiological values suitable for cell growth.

**Figure 9:**
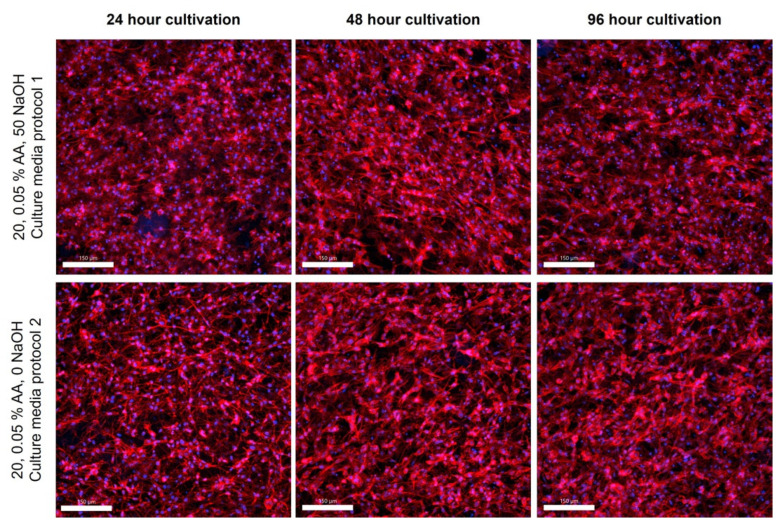
Confocal microscopy images of cultivated samples in 20 mg/mL collagen dissolved in 0.05 wt.% acetic acid prepared using two different culture media (protocol 1, e.g., pH adjusted culture medium, and protocol 2, culture medium with no pH adjustment). Cultivation for 24, 48, and 96 h. F-actin was stained using Alexa Fluor™ 594 Phalloidin (red), and cell nuclei were counterstained using DAPI (blue), scale 150 µm. Nikon CSU-W1 confocal microscope with CFI Plan Apo VC 20× dry objective. Orthographic projection rendered.

A comparison of two bioink production methods (Figure 9) where 10× concentrated culture medium was (un)adjusted to pH 7.0 before mixing also showed no differences in cell morphology and proliferation. After 96 h of culture, the cells were viable, elongated, and formed a structure similar to that of physiological tissue.

**Figure 10:**
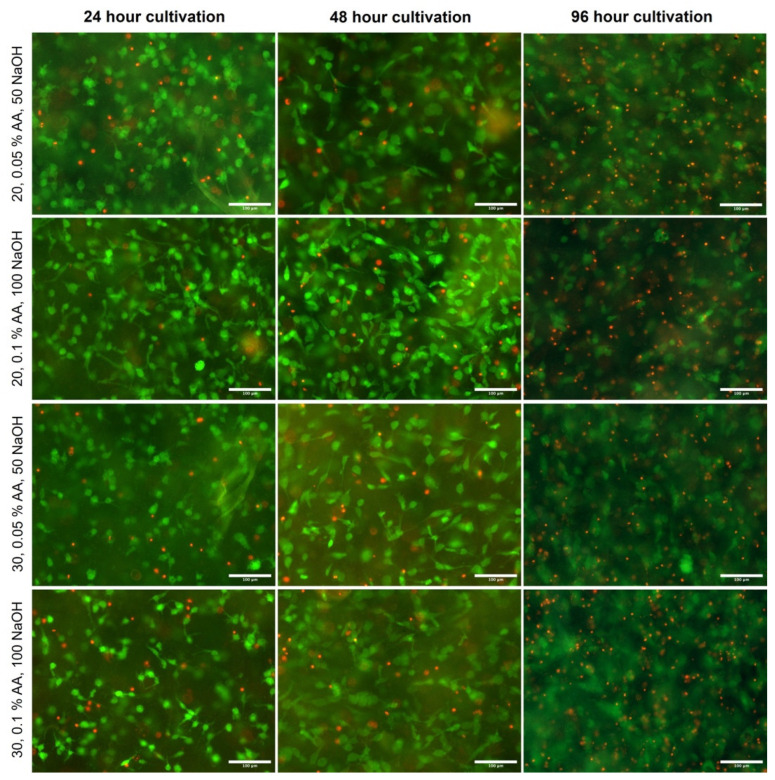
Live/dead images of cultivated samples in 20 and 30 mg/mL collagen dissolved in 0.05 and 0.1 wt.% acetic acid. Cultivation for 24, 48, and 96 h. Fluorescein diacetate-stained live cells (green) and dead cells (nuclei) stained with propidium iodide (red), scale 100 µm. Custom-built Thorlabs CERNA fluorescence microscope with Olympus Plan Fluorite 10× objective.

**Figure 11:**
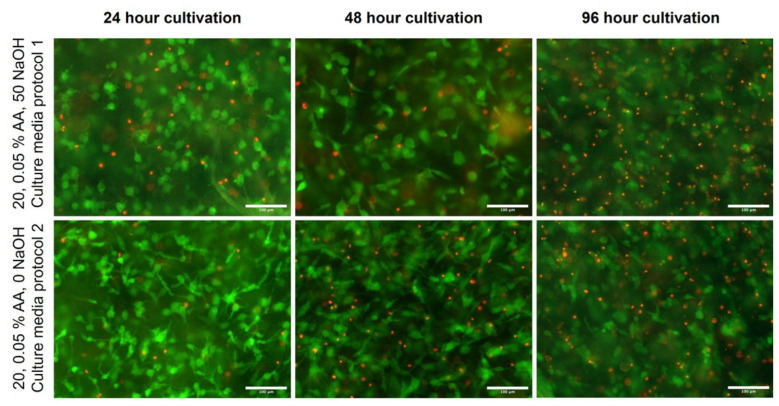
Live/dead images of cultivated samples in 20 mg/mL collagen dissolved in 0.05 wt.% acetic acid were prepared using two different culture media (protocol 1, e.g., pH adjusted culture medium, and protocol 2, culture medium with no pH adjustment). Fluorescein diacetate-stained live cells (green) and dead cells (nuclei) stained with propidium iodide (red), scale 100 µm. Custom-built Thorlabs CERNA fluorescence microscope with Olympus Plan Fluorite 10× objective.

However, unlike cell morphology, cell viability differed between samples (Figure 10). The highest viability was observed for the 20 mg/mL, 0.1% AA, 100 NaOH sample and the 30 mg/mL, 0.05% and 50 NaOH samples; the other two samples showed higher cell mortality, which was obviously evident at other times. However, the comparison of multiple samples showed that the differences between samples were not statistically significant because the pH neutralization of the collagen hydrogels was emphasized in the preparation of the bioinks. Regardless of the initial concentration of collagen and acetic acid, the resulting bioink is chemically and structurally similar in all samples, and thus differences in cell growth and viability are not apparent. The comparison of the two production methods again showed small differences, especially after 24 h of cultivation, which were again not statistically significant (Figure 11). Several factors influence cell viability, particularly in 3D samples: the actual structure, homogeneity, and thickness of the layer in which the cells are incorporated. The higher density of collagen fibers can be concentrated in one location, preventing diffusion, and cells are not optimally nourished. Furthermore, the viability is affected by random effects during bioprinting, the temperature acting on the cells, the mechanical pressure, and the chemical effects of the components. After 96 h of culture, the images show the highest proportion of dead cells. As a result of the growth of cells throughout the gel volume, dead cells cannot be washed out during medium exchange, as in flat culture on the substrate. Thus, these cells accumulate there, and viability seems to be lower than in previous intervals. Also, wide-field fluorescence microscopy has its limits in taking images, especially in 96 h intervals from gel substrates. It is also expected that cells partially remodel the substrate. In images from confocal microscopy, there were no significant changes in the morphology or structure of cells in the 96 h interval compared to 48 h. Also, a limitation may be the substrate used (coverslip glass) that limits diffusion from one side of the 3D construct. The use of a mild culture perfusion in a bioreactor can also improve these problems.

### 2.7. Benefits and Risks of Current Bioink Formulation

The formulation of bioink in our previous work [23] was optimized to acquire the most suitable pH for the neutralization of the collagen hydrogel to promote its gelation, resulting in a stable bioink for 3D bioprinting and a printed substrate with cells. However, this formulation, which modifies the resulting pH, lacks nutrients and uses extremely low volumes of cell suspension, resulting in a complicated preparation.

In this study, we have modified our bioink based on a highly concentrated collagen hydrogel (20 and 30 mg/mL). Optimization of the formulation, especially in terms of FBS-derived growth factors, resulted in improved cell culture viability and cell growth and division during the 4-day period studied. Furthermore, the change in the volume ratio of the different bioink components led to the fact that the mixing process could be automated, which not only speeds up the process but also continuously monitors the pH during the bioink production process. Automated mixing also reduces the temperature shock to cells during bioink formation, which can negatively affect cell viability. The new method based on highly concentrated collagen may be a promising 3D bioprinting bioink for use in tissue engineering.

On the other hand, the cultivation of 3D scaffolds is practically limited by the thickness of the scaffold, with simple diffusion transferring nutrients and oxygen and removing waste products only for structures up to a maximum thickness of 2 mm. To nourish cells to deeper layers, a transport system, e.g., channels, must be added to permeate the entire volume of the scaffold. However, the use of type I collagen for bioprinting requires further research. In fact, some studies have shown that its use induces cell dedifferentiation, as demonstrated, for example, in chondrocytes in the study by Farjanel et al. [28]. The rapid expansion of chondrocytes in the monolayer leads to their dedifferentiation [29]. Furthermore, the synthesis of type III collagen to type I collagen occurred [29].

## 3. Conclusions

This study investigated the ability to control the collagen neutralization process that provides a printable bioink. These steps were utilized by our custom-built semiautomatic mixing system. The collagens used in this study were in two concentrations of 20 and 30 mg/mL dissolved in two concentrations of acetic acid 0.1 and 0.05 wt.%. In addition, phenol red was added to the dissolved collagen hydrogels, allowing colorimetric estimation of pH. To neutralize collagen hydrogels, a new formulation of culture media compositions was made—2× enhanced. A mixing ratio of 2:1:1 (collagen hydrogel:neutralization medium: cell suspension) was established as optimal to provide sufficient volumes for neutralization and the addition of cell suspension. The prepared collagens were titrated to determine the optimal volume of medium to neutralize the pH of the collagens by a bicarbonate buffering mechanism. Therefore, the method of 2 neutralizations was established to balance the pH of collagen hydrogels by supplying 0–100 uL/mL NaOH. After the first neutralization, the medium would be suitable for the addition of the cell suspension that constitutes the second neutralization. The amount of NaOH added cannot be fixed; it always depends on circumstances such as the buffering capacity of the culture medium, the pH of the batch of collagen hydrogels, etc. However, based on the established methodology of 2 neutralizations, the collagen bioinks were verified by 96 h culture. Cell viability and proliferation were not reduced after 96 h of culture.

## 4. Materials and Methods

### 4.1. Collagen Isolation

Type I collagen (COL), which was used for the preparation of hydrogels, was isolated from porcine skin according to our previous study [23]. Briefly, degreasing procedures were performed using 70 vol.% ethanol solution followed by the extraction of COL by application of 0.1 vol.% acetic acid solution (AA). After centrifugation, the COL in the collected supernatant was precipitated using a 0.1 M NaOH solution up to neutral pH. The obtained COL pellets after centrifugation were then dissolved in 0.1 vol.% acetic acid solution, frozen to −30 °C, and lyophilized. All isolates were stored in a freezer at −20 °C.

### 4.2. Preparation of Collagen Hydrogels

Lyophilized COL I was sterilized by immersion in 70 vol.% ethanol for 2 h. After removal from ethanol, the COL was freely dried in a laminar flow box. COL was then dissolved in 0.05 wt.% and in 0.1 wt.% acetic acid in a COL concentration of 20 mg/mL and was stored at 4 °C for 10 days. Phenol red in the amount of 0.008 g was added to 1 L of acetic acid solutions to observe the changes in pH during further operations. The COL suspension was further homogenized using a disintegrator with 10,000 rpm for 2 min (IKA Dispersers, T 10 basic ULTRA-TURRAX^®^, Staufen, Germany) to obtain a homogenous COL solution.

### 4.3. Collagen Hydrogel Preparation

Lyophilized COL I was sterilized by immersion in 70 vol.% ethanol for 2 h. After removal from ethanol, the COL was dried freely in a laminar flow box. The COL was then dissolved in 0.05 wt.% and 0.1 wt.% AA in a concentration of COL of 20 or 30 mg/mL and stored at 4 °C for 10 days. Phenol red in the amount of 0.008 g was added to 1 L of AA solutions to observe changes in pH during future operations. The COL suspension was further homogenized using a disintegrator with 10,000 rpm for 2 min (IKA Dispersers, T 10 basic ULTRA-TURRAX^®^, Staufen, Germany) to obtain a homogenous COL solution.

### 4.4. Analysis of Porcine Collagen Properties

The secondary structure COL lyophilizates (original and dissolved in both concentrations of acetic acid) were evaluated by attenuated total reflection infrared spectrometry (ATR-FTIR) using an iS50 infrared spectrometer (Nicolet Instrument, Madison, WI, USA), the ATR device was equipped with a diamond crystal. Spectra were scanned in absorption mode in the range 4000–400 cm^−1^ at a resolution of 4 cm^−1^ 64 times. Infrared spectra were processed and evaluated using the OMNIC version 9 software. The spectra of studied samples were compared with commercial COL (lyophilized rat tail collagen; ROCHE, Basel, Switzerland), and they were scanned in a lyophilizate state several times to verify the homogeneity of the material.

### 4.5. Composition of Culture Media Used for Neutralization of Collagen Hydrogel

In the experiments, the control samples prepared according to the method of our previous work [23] and the samples prepared according to the new method were compared. For the current experiments, the culture medium for the bioink preparation was enriched with its components so that the resulting concentration of all components in the bioink (i.e., after mixing with collagen and cell suspension) was 1× that of the standard growth medium for stem cell culture, except for fetal bovine serum, which is 10 times more concentrated (in the original formulation only 3× more concentrated), as shown in Table 1.

The original culture growth medium (marked in the table as DMEM/F12 FBS FGF ABAM) is composed of a 1:1 mixture of Dulbecco’s modified Eagle Medium and Ham’s F-12 Medium (DMEM:F12, Gibco; supplemented with 2.438 g/L sodium bicarbonate), with 10% fetal bovine serum, 1% ABAM antibiotics (100 IU/mL penicillin, 100 µg/mL of streptomycin, and 0.25 µg/mL of Gibco Amphotericin B; Sigma-Aldrich, St. Louis, MO, USA) and 10 ng/mL FGF2.

Ten times (10×) concentrated DMEM/F12 was prepared using two protocols, differing in pH adjustment. The manufacturer’s recommendations for 1× concentrated medium were followed in the preparation of the media. The medium prepared according to protocol 1 contained 1/10 water, and the pH of the medium was adjusted to pH 7.0 using HCl. Then, the medium was sterilized. In protocol 2, no HCl was added, and the pH of the medium was left at 7.5 and then filtered.

### 4.6. Cell Survival in Newly Prepared and Concentrated Media

The tests were performed in various cell concentrations in compensated and uncompensated culture medium and compared to standard proliferation media. Culture media were prepared according to Table 1. Culture media were tested with cells in which collagen was replaced with dH_2_O. Cells were seeded in culture wells at a concentration of 10, 50, and 100,000 cells per cm^2^. Cell viability was evaluated 3 days after seeding compared to the conventional growth medium.

Porcine stromal cells derived from porcine adipose tissue were used for all the experiments in this article. The entire procedure, including cell characterization, is described in a previous article by Matejka et al. [30]. Cells were harvested in 3–5 passages after cultivation in a cell growth medium. After centrifugation, cells were resuspended in a 2× enhanced culture medium.

### 4.7. Medium Preconditioning

The culture media used in this study use a bicarbonate buffer system with a 5% CO_2_ atmosphere to maintain optimal pH. When fresh culture media is prepared, its optimal pH is in the physiological range of 7.2–7.4. However, manipulation in a normal atmosphere, especially in small volumes, tends to change the pH of the medium to alkaline [31]. Furthermore, in our case, we have prepared ‘2× enhanced’ culture media consisting of twice bicarbonate compared to normal culture media. This increased bicarbonate shifts the prepared medium above the physiological range of 7.5–7.8 (according to the protocol used) and is more prone to change pH to alkaline in an atmosphere without CO_2_.

**Figure 12:**
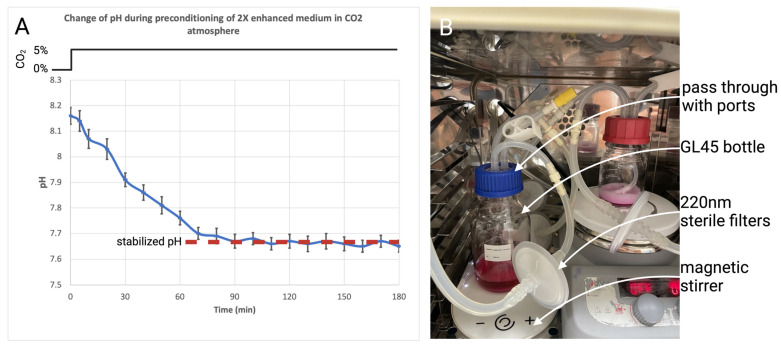
(**A**) Change in pH during preconditioning in CO_2_ atmosphere; (**B**) setup for preconditioning of sterile culture medium.

To achieve an optimized pH of the culture medium used to mix the printable bioink that shifts the pH to the base, we have constructed a preconditioning setup. This setup consists of a standard GL45 bottle with a custom 3D printed pass-through plug with three fluidic ports. Two ports are used to recirculate the CO_2_ atmosphere through two 220 nm PTFE sterile filters. The third port allows for the aseptic connection of a syringe to obtain a pH-optimized medium. When the medium is placed in the syringe without any air gaps, the change in pH is minimal. To speed up the process, a magnetic stirrer was also used. According to pH measurements and depending on volume, the culture medium stabilized to pH 7.6–7.7 in 120 min. This setup is illustrated in Figure 12.

### 4.8. Preparation of Printable Bioinks

In this study, we have formulated an optimal mixing ratio of 2:1:1 (collagen hydrogel:neutralization medium:cell suspension medium). This ratio was chosen to achieve a high concentration of the resulting collagen, the ability to control the pH of the resulting bioink using the neutralization step, and the ability to prepare cell suspensions with densities lower than in our previous work [23].

**Figure 13:**
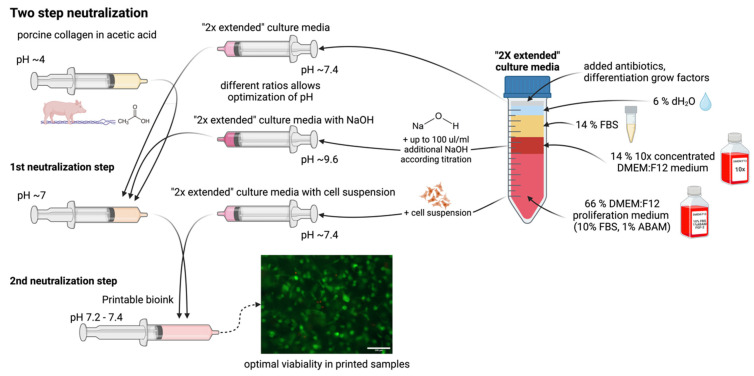
Schematic process of preparation of “2× enhanced” culture medium with its modification and two-step neutralization mixing procedure for preparation printable bioink.

The preparation of the printable bioink is performed in two steps (two-step neutralization). In the first step, collagen gel dissolved in acetic acid (AA) is mixed with a neutralization medium. This neutralization medium is based on 2× enhanced culture media and can be mixed with the same medium with added NaOH. This step is crucial to neutralize acidic collagen to a neutral pH of around 7. In the second step, the cell suspension in the 2× enhanced medium is mixed into the pre-neutralized solution, which transforms into the final bioink. Throughout the process, the syringes containing all components are cooled to 4 °C to prevent premature gelation. These procedures are schematically shown in Figure 13.

To test the pH trend in the bioink, three different concentrations of 1 M NaOH were added to the 2× enhanced medium to form the neutralization medium: 0, 50, and 100 μL/mL. Four groups of samples were prepared, varying in collagen concentration and solvent concentration: 20 mg/mL and 0.1% AA, 30 mg/mL and 0.1% AA, 20 mg/mL and 0.05% AA, and finally 30 mg/mL and 0.05% AA. During the preparation of the bioinks, the pH of all components was measured colorimetrically. These initial tests to determine the optimal pH were performed without cell addition.

**Figure 14:**
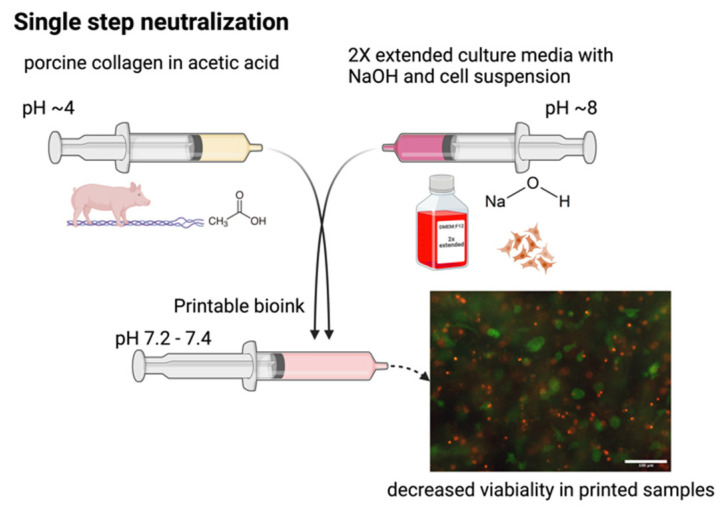
The schematic process of single-step neutralization provides poor results in cell viability.

We have also tested the possibility of mixing neutralization and cell suspension media and then mixing them with collagen. There were similar results with the resulting pH of the bioink; however, cell viability was decreased as a result of the high pH in the culture medium, and the large difference was decreased. This procedure, called ‘single-step neutralization’, is illustrated in Figure 14 and was not used in the preparation process.

### 4.9. Colorimetric pH Evaluation

The estimation of pH is crucial in the collagen neutralization process. First, it influences cell viability, and second, it affects printing properties [23]. Estimation with a conventional pH probe or touch pH probe has its limitations, which involve a long settling time, unsterile measurements, and a large sample volume. However, culture media and our prepared collagen hydrogels also contain phenol red as a pH indicator, allowing colorimetric estimation. For this purpose, we have created a unique cooled syringe holder equipped with a colorimetric sensor an EZO-RGB™ Embedded Color Sensor (Atlas Scientific, NY, USA). This colorimetric sensor provides output in the standardized CIE 1931 color space with outputs of x, y, and Y, where x and y are color space coordinates and Y is luminance. The white-point calibration of the colorimeter was set to the standardized D65 illuminant. This white point estimation was performed on the white and gray card, and then a syringe with white silicone was used. Calibration of pH was performed for both culture mediums (control, extended) and both collagen hydrogels. The standard flat-head touch electrode was used as a reference (THETA 90, Prague, Czech Republic).

**Figure 15:**
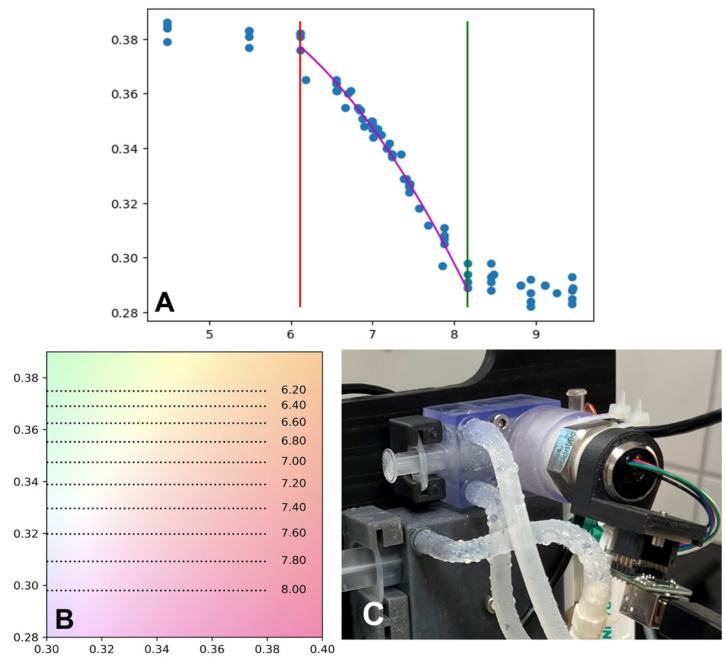
(**A**) calibrating values for estimation of pH from CIE-y coordinate shown by blue dots, red and green line define the measurable pH response of phenol red (**B**) pH scale over a section of CIE 1931 diagram, (**C**) colorimeter in mixing syringe-cooled block.

The most important factor in determining pH was the CIE-y value, which changes in the pH range from 6.2 to 8.1. The measured data were fitted using a parabolic curve, and these values were also placed on a section of the CIE diagram. The measured CIE-x and Y values were affected by the type of media and hydrogel; however, they were not affected by the pH. These processing steps are illustrated in Figure 15.

A major advantage of this approach is the fast response and measurement over the syringe wall, which ensures sterility. Based on fitted data and reverse evaluation, the resolution of the setup is better than 0.1 pH. The following measurements were made with this colorimetric setup.

### 4.10. Semiautomated System for Bioink Preparation

As mentioned above, the preparation of printable collagen bioink utilizes two steps: collagen hydrogel is first neutralized to a pH of around 7, and then cell suspension is added. In these procedures, the collagen hydrogel needs to be precisely dosed, and then a 2× enhanced culture medium with optimized pH is added. These components are dosed in hundreds of microliter volumes. Even small changes in volume can extensively affect the pH, printing, and gelling parameters of the prepared collagen bioink. Due to the low volumes needed and repeatability, we have constructed a custom semiautomated mixing system.

This system consists of three motorized linear actuators that hold three 10 mL syringes containing collagen, 2× enhanced culture medium, and 2× enhanced culture medium with the addition of 100 µL/mL NaOH. These three syringes are connected to a five-element, three-way stopper manifold. The upper two ports of the manifold connect the manual mixing syringe and the printhead syringe. All syringes are kept cooled below 10 °C by circulating water. The cooled mixing syringe holder also contains a colorimeter. The manifold is cleaned using sterile PBS and a connection to a vacuum.

**Figure 16:**
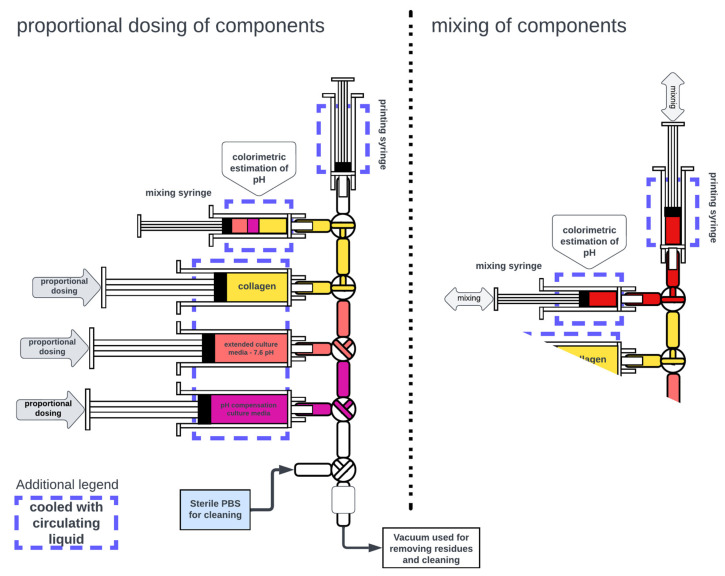
Schematic of the semiautomated mixing system.

**Figure 17:**
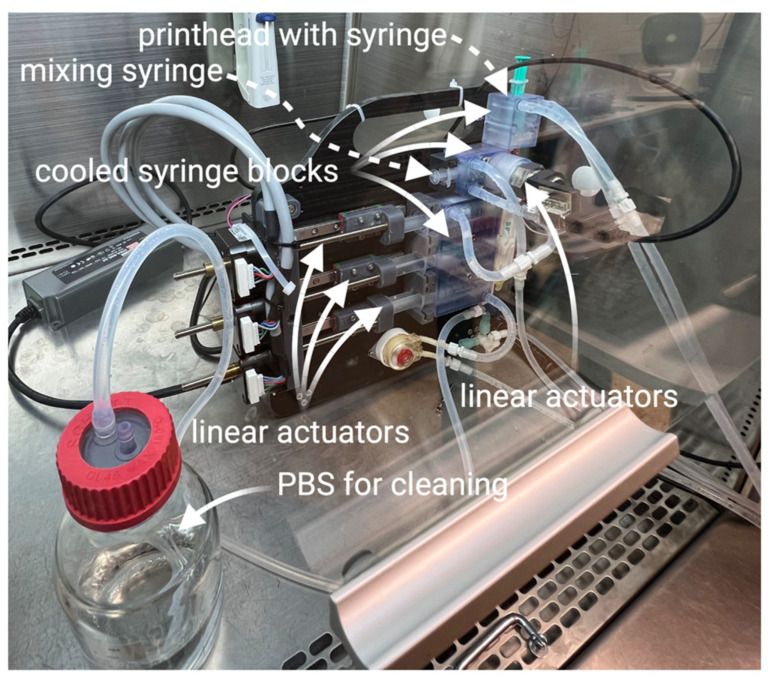
Mixing system installed in the biohazard box.

The preparation of the bioink in this system consists of several steps. First, a neutralization medium with a given pH (added NaOH concentration) was prepared. This step consists of dosing the desired volumes of extended and NaOH-enhanced culture media from cooled syringes. In this study, we used fixed steps of NaOH concentrations (0, 25, 50, and 100). However, the system allows for proportional mixing and titration. The next steps utilize the dosing of collagen in the prepared medium, followed by manual mixing. This mixing is performed by a repeated manual push of the mixing and printing syringe plungers. A schematic of the semiautomated system with its components is illustrated in Figure 16 and Figure 17.

### 4.11. Bioprinting

Five groups of samples were printed, varying in collagen concentration and solvent concentration, and modified neutralization culture media: 20 mg/mL and 0.1% AA with 100 μL/mL neutralization medium, 30 mg/mL and 0.1% AA with 100 μL/mL neutralization medium, 20 mg/mL and 0.05% AA 50 μL/mL neutralization medium, 30 mg/mL and 0.05% AA 50 μL/mL neutralization medium and finally 20 mg/mL and 0.05% AA with medium protocol 2 without added NaOH in neutralization medium. The final cell density was set to 10 mil. cells/1 mL of bioink.

**Figure 18:**
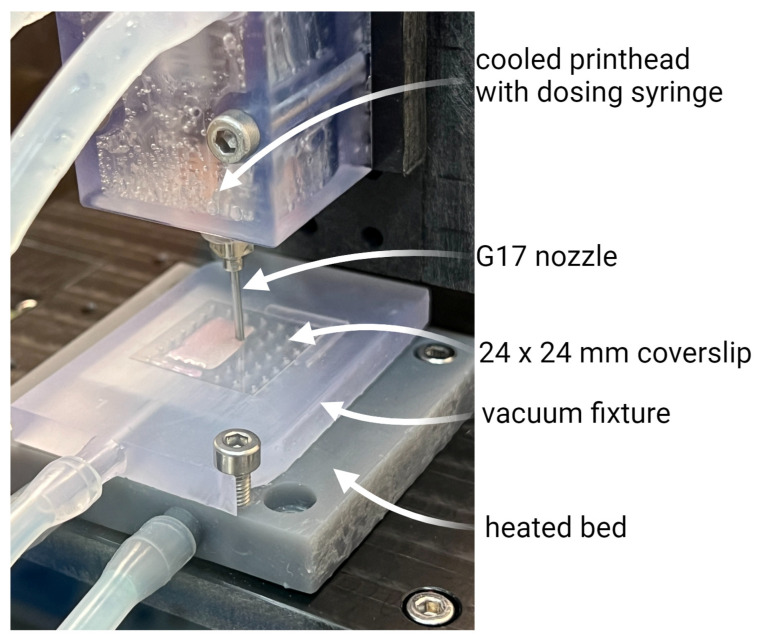
Bioprinting on 24 × 24 mm coverslip glass.

The prints were made in our modified bioprinter on tissue-treated coverslip glass (24 × 24 mm). The 15 × 15 × 1 mm square was set as a printing shape. Slicing was set to two layers with a G17 nozzle. The printing feed rate was set to 300 mm/min. The printing head was kept cool at <10 °C using cooled circulating water, while the print bed with the vacuum fixture was heated to 37 °C using heated circulating water. The print setup is illustrated in Figure 18.

Printed samples were placed in six-well plates and flooded with culture media. The culture was held for 24, 48, and 96 h. Each culture interval was microscopically evaluated for cell viability and morphology.

### 4.12. Cell Viability Analysis

Cell viability was identified by live cell staining with fluorescein diacetate (FDA, 5 mg/mL; F1303, ThermoFisher, MA, USA) and dead cell staining with propidium iodide (PI, 2 mg/mL; P1304MP, ThermoFisher, MA, USA) after 24, 48 and 96 h of culture of printed samples. Samples were rinsed three times with PBS and stained with FDA and PI diluted in culture medium without fetal bovine serum for 5 min at 37 °C in a humidified incubator. According to the manufacturer’s instructions, live and dead cells were labeled green and red. The images were then taken using a custom-built Thorlabs CERNA (Thorlabs, Bergkirchen, Germany) wide-field fluorescence microscope with Olympus Plan Fluorite 10× objectives (Olympus, Tokyo, Japan) and DCU223M CCD camera (The Imaging Source, Bremen, Germany).

### 4.13. Cell Morphology

The filamentous actin (F-actin) in the cell cytoskeleton was stained using Alexa Fluor™ 594 Phalloidin (Thermo-Fisher, Waltham, MA, USA, Cat. No. A12381, 200 U/mL) and cell nuclei using DAPI (Thermo-Fisher, D1306, 300 nM concentration). Staining was performed in humidified chambers for 120 min at room temperature. After staining, the samples were rinsed and stored in PBS. Confocal microscopic images were taken using a Nikon CSU-W1 inverted spinning disc confocal microscope based on the Nikon Eclipse Ti2 inverted microscope (Nikon, Tokyo, Japan) with the Yokogawa CSU-W1 spinning disc module (Yokogawa, Tokyo, Japan) and equipped with dual sCMOS PRIME BSI cameras (Teledyne Photometrics, Tucson, AZ, USA). Due to the manner of the samples, Nikon dry objectives CFI Plan Apo VC 20x were used with a 50 mm pinhole disc in Z stack mode (–0–250 mm depth). Images were then processed in Imaris 10.1.0 software (Oxford Instruments, Abingdon-on-Thames, UK).

## Figures and Tables

**Table 1:** Comparison of the composition culture medium prepared according to the previous protocol and the new protocol.

	Medium Composition According to [23]			New Composition		
		Partial Concentration (per 1 mL of Culture Media)			Partial Concentration (per 1 mL of Culture Media)	
	Volume Concentration	Ionic (DMEM:F12)	Nutrients (FBS)	Volume Concentration	Ionic (DMEM:F12)	Nutrients (FBS)
10× DMEM/F12	3.5%	0.7	0.0	3.5%	0.70	0.00
DMEM/F12 FBS FGF ABAM	16.5%	0.3	3.3	16.5%	0.33	3.30
H_2_O	30.0%	0.0	0.0	26.5%	0.00	0.00
FBS	0.0%	0.0	0.0	3.5%	0.00	7.00
Concentration without collagen	50.0%	2.1	6.6	50%	2.06	20.60
Collagen	50.0%	0.0	0.0	50%	0.00	0.00
Resulting concentrtion	100.0%	1.03	3.3	100%	1.03	10.3

## Data Availability

All data and materials are available on request from the corresponding author. The data are not publicly available due to ongoing research using a part of the data.
